# The Effect of Naphthazarin on the Growth, Electrogenicity, Oxidative Stress, and Microtubule Array in *Z. mays* Coleoptile Cells Treated With IAA

**DOI:** 10.3389/fpls.2018.01940

**Published:** 2019-01-08

**Authors:** Małgorzata Rudnicka, Michał Ludynia, Waldemar Karcz

**Affiliations:** Department of Plant Physiology, Faculty of Biology and Environmental Protection, University of Silesia, Katowice, Poland

**Keywords:** naphthazarin, IAA, maize, growth, oxidative stress

## Abstract

Naphthazarin (5,8-dihydroxy-1,4-naphthoquinone, DHNQ) is a naturally occurring 1,4-naphthoquinone derivative. In this study, we focused on elucidating the toxic effect of this secondary metabolite on the growth of plant cells. The dose–response curves that were obtained for the effects of DHNQ on endogenous and IAA-induced growth in maize coleoptile segments differ in shape; in the first case, it is linear, while in the presence of auxin it is bell-shaped with the maximum at 1 μM. It was found that DHNQ at almost all concentrations studied, when added to the incubation medium inhibited endogenous growth (excluding naphthazarin at 0.001 μM) as well as growth in the presence of IAA. Simultaneous measurements of the growth and external medium pH of coleoptile segments indicated that DHNQ diminished or eliminated proton extrusion at all of the concentrations that were used. Interestingly, the oxidative stress in maize coleoptile cells, which was measured as hydrogen peroxide (H_2_O_2_) production, catalase activity, redox activity and malondialdehyde (MDA) content, increased at the lower concentrations of DHNQ (<1 μM), thus suggesting a specific character of its action. It was also found that naphthazarin at concentration higher than 0.1 μM caused the depolarization of the membrane potential (*E*_m_). An analysis of the organization and anisotropy of the cortical microtubules showed that naphthazarin at all of the concentrations that were studied changed the IAA-induced transverse microtubule reorientation to an oblique reorientation. Our results indicate that naphthazarin diminished the growth of maize coleoptile cells by a broad spectrum of its toxic effects, thereby suggesting that naphthazarin might be a hypothetical component of new bioherbicides and biopesticides.

## Introduction

Naphthoquinones are the products of the bacterial and fungal metabolism as well as the secondary metabolism in higher plants, where they are produced and used as natural defense chemicals (Babula et al., 2009; War et al., 2012). Among these natural products, 1,4-naphthoquinone derivatives such as juglone (5-hydroxy-1,4-naphthoquinone), lawsone (2-hydroxy-1,4-naphthoquinone), plumbagin (2-metyl-5-hydroxy-1,4-naphthoquinone), naphthazarin (5,8-dihydroxy-1,4-naphthoquinone) and others along with their natural and synthetic derivatives have been studied in biology and medicine for some time. Currently, the unique properties of naphthoquinones are of the focus of interest within industry; for example, 2-hydroxy-1,4-naphthoquinone and 1,4-naphthoquinone can be used as corrosion inhibitors for mild steel and aluminum (Sherif and Park, 2006; Ostovari et al., 2009). Naphthazarin is one of the natural 1,4-naphthoquinone substances that are derived from the tissues of several members of the *Boraginaceae, Droseraceae*, and *Nepenthaceae* families (Papageorgiou et al., 1999; Devi et al., 2016). The substances that are produced by the secondary metabolism pathways probably play a protective role as a chemical defense against herbivores. Among the substances that have such defensive functions, phenols, lignins, flavonoids, tannins, and quinones should be mentioned. It has been found that quinones, *via* the alkylation of proteins or interactions with other organic molecules, reduce the nutritional value of plant components for insects, thereby leading to a reduction in their growth and development. Secondary metabolites, such as quinones and their derivatives, may also have a direct toxic effect on insect-attacking plants as a result of the initiation of the redox cycles and the production of reactive oxygen species (ROS). Interestingly, it was also found that quinone derivatives are primarily synthesized during the stress that is associated with a herbivorous attack (Duffey and Stout, 1996; Eilenberg and Zilberstein, 2008; War et al., 2012).

Some of the naphthazarin toxins such as dihydrofusarubin, marticin, isomarticin, and methyl javanicine, which are produced by *Fusarium solani*, can affect higher plants, and the toxins that are most efficacious to plants are marticin and isomarticin (Kern and Naef-Roth, 1967; Kern, 1978; Marcinkowska, 1981; Baker and Tatum, 1983). It has been shown that two of the naphthazarin phytotoxins, dihydrofusarubin and isomarticin, affected the permeability of citrus leaf tissue, reduced water uptake in intact seedlings and stimulated vessel plugging in seedling rootstocks that was maintained in dilute toxin solutions (Nemec et al., 1988). In later studies with both phytotoxins, it was shown that they also affected the organellar membranes, primarily those of the chloroplasts, plasmalemma and tonoplast (Achor et al., 1993). Moreover, Albrecht et al. (1998) reported that tobacco leaves that had been incubated with dihydrofusarubin had a light-dependent degradation of leaf pigments and that dihydrofusarubin interacted with the photosynthetic electron transport chain of spinach chloroplasts, thus forming ROS.

Naphthazarin and its derivatives have a wide variety of pharmacological activities, including anticancer, anti-inflammatory, antibacterial and antifungal effects (Sasaki et al., 2002; Shen et al., 2002; Kim et al., 2015; Zhang et al., 2017). Due to their properties, naphthoquinones have become an object of interest in agriculture which, according to recent pro-ecological trends, has put emphasis on the use of substances that naturally occur in the environment. Naphthoquinones and other plant secondary metabolites can potentially be used as biopesticides and bioherbicides due to the multiplicity of their modes of action (MOAs) compared to traditional plant protection products (Dayan and Duke, 2014). It was found that secondary metabolites such as juglone or citral exhibit a high phytotoxicity by affecting the plasmalemmal H^+^-ATPase activity or the polymerization of microtubules (Hejl and Koster, 2004; Chaimovitsh et al., 2010).

In addition, naphthazarin has high redox properties and can be used as an organic component of the positive electrode in an ecological battery (Yao et al., 2017).

In this study, as was the case in our earlier investigation (Rudnicka et al., 2014, 2018), we chose the elongation growth of maize coleoptile segments as the main parameter in order to assess the biological activity of naphthazarin. Two facts should be added here: (1) that the coleoptile of grasses represent a classical model system for studies on the elongation growth of plant cells in which the number of cells is constant and the organ grows only *via* elongation (see Kutschera and Wang, 2016) and (2) that most of crucial evidence on the mechanisms of auxin action in plant cell growth was obtained from grass coleoptile segments (reviewed in Rayle and Cleland, 1992; Schopfer, 2001; Hager, 2003). In addition to elongation growth, the medium pH changes of coleoptile segments (measured simultaneously with growth) and the membrane potential of the cells were also determined. The relationships between these parameters are fundamental for the so-called “acid growth hypothesis” of auxin-induced growth (for a review, see Rayle and Cleland, 1992; Hager, 2003). Moreover, H_2_O_2_ production, catalase (CAT) activity, MDA content and the organization of the microtubules were also found.

Here, we focused on elucidating the toxic effect of naphthazarin (5,8-dihydroxy-1,4-naphthoquinone, DHNQ) on the growth of plant cells. This goal was achieved by (1) studying the effect of naphthazarin on the endogenous and IAA-induced growth of maize coleoptile segments and the medium pH, which was measured simultaneously with growth; (2) determining the impact of naphthazarin on changes in the membrane potential in the parenchymal cells of maize coleoptile segments that had been incubated in the presence and absence of IAA; (3) establishing the influence of DHNQ on H_2_O_2_ production and CAT activity in coleoptile segments; (4) studying the impact of DHNQ on the plasma membrane redox activity; (5) examining the effect of DHNQ on the malondialdehyde (MDA) content of coleoptile segments that had been incubated with or without IAA; and (6) studying the effect of naphthazarin on the organization of the microtubules. To the best of our knowledge, naphthazarin has never been examined for its ability to regulate auxin (IAA)-induced growth and electrogenic activity of plant cells. This experimental design can provide new data on the effects of naphthazarin on plant growth.

## Materials and Methods

### Plant Material

Caryopses of maize (*Zea mays* L. cv. Cosmo 230) were soaked in tap water for 2 h, sown on wet lignin in plastic boxes and placed in a growth chamber (Type MIR-553, Sanyo Electric Co., Osaka, Japan) at 27 ± 1.0°C for 4 days in darkness. The experiments were performed on ten-mm-long coleoptile segments that had been cut from maize etiolated seedlings (length of the coleoptile 2–3 cm). The coleoptile segments with the first leaves removed were excised 3 mm below the tip and collected in a control medium comprising 1 mM KCl, 0.1 mM NaCl and 0.1 mM CaCl_2_. In all of the growth experiments, the initial pH of the control medium was adjusted to 5.8–6.0. Conditions for growing the maize seedlings and cutting of coleoptile segments have been described previously (Kutchera and Schopfer, 1985; Lüthen et al., 1990; Karcz and Burdach, 2002; Burdach et al., 2014).

### Growth and pH Measurements

The growth experiments on the coleoptile segments were carried out in an apparatus that enabled the simultaneous measurements of the elongation growth and the pH of the incubation medium from the same tissue sample (Polak et al., 2012; Burdach et al., 2014; Rudnicka et al., 2018). Briefly, 60 coleoptile segments were arranged vertically in three narrow glass pipettes (20 segments in each), which were connected in this apparatus using a silicon hose. The medium was circulated by a peristaltic pump (1B-05A; Zalimp, Warsaw, Poland). High-resolution measurements of the growth rate were performed using an angular position transducer (TWK-Electronik, Düsseldorf, Germany). The coleoptile segments were incubated in an intensively aerated medium in which the volume of the incubation medium in the elongation- and pH-measuring apparatus was constant (0.3 ml/segment). The incubation medium also flowed through the lumen of the coleoptile cylinders. This feature enabled the experimental solutions to be in direct contact with the interior of the segments, which significantly enhances both the elongation growth of the coleoptile segments and proton extrusion (Karcz et al., 1995). The extension growth of a stack of 20 segments and the pH of the incubation medium were sampled every 3 min using a multifunctional computer meter (CX-771; Elmetron, Zabrze, Poland). The pH measurements were performed with a pH electrode (OSH 10-10; Metron, Torun, Poland). All of the manipulations, growth and pH measurements were carried out under dim green light at a thermostatically controlled temperature of 25 ± 0.5°C. IAA was used at a final concentration of 100 μM. This concentration is optimal for the elongation growth of the maize coleoptile segments, which was measured over 10 h in our elongation- and pH-measuring apparatus (Polak, 2010). For comparison, IAA at 10 μM is optimal at the same experimental conditions, however, in short-term recordings (Karcz et al., 1990; Karcz et al., 1999).

### Electrophysiology

The electrophysiological experiments were performed on coleoptile segments that were prepared in the same manner as for the growth experiments. A standard electrophysiological technique was used for the membrane potential measurements as was previously described by Karcz and Burdach (2002) and Burdach et al. (2014). Briefly, the membrane potential (*E*_m_) was measured by recording the voltage between a 3 M KCl-filled glass micropipette that was inserted into the parenchymal cells and a reference electrode in the incubation medium of the same composition as the one that was used in the growth experiments. For the electrophysiological experiments, the segments were preincubated for 1 h in an intensively aerated control medium, after which the segments were transferred into a perfusion Plexiglas chamber (containing the control medium), that was mounted on a vertically placed microscope stage. After insertion of the microelectrode into the cell and stabilization of *E*_m_ (<10 min) (time “0” in the electrophysiological experiments) the control medium or medium with DHNQ was exchanged for a new one, depending on the variant of the experiment. The medium changes were performed using a peristaltic pump (Peri-Star PRO; World Precision Instruments, Sarasota, FL, United States) after the stabilization of the membrane potential (*E*_m_). This type of peristaltic pump permits the incubation medium in the chamber to be changed (usually four times within less than 2 min) without any visible disruption of the measurements. It should be also added that the peristaltic pump was used only during exchange of the medium. The microelectrodes were inserted into the cells under a microscope using a micromanipulator (Hugo Sach Elektronik; MarchHugstteten, Germany). The micropipettes were made from borosilicate glass capillaries (type 1B150F-3; World Precision Instruments, Sarasota, FL, United States) using a vertical pipette puller (Model PIP 6; HEKA Elektronik, Lambrecht, Germany).

### Hydrogen Peroxide Detection

The hydrogen peroxide (H_2_O_2_) concentration in all of the variants that were tested was determined according to Velikova et al. (2000) with minor modifications. Coleoptile segments were preincubated for 1 h in control medium and immediately transferred to control medium with DHNQ at the appropriate concentration. IAA, at a final concentration of 100 μM, was included in the incubation medium when required. Briefly, after appropriate time of incubation 0.5 g coleoptile segment samples were homogenized in 1.5 ml of 0.1% (w/v) trichloroacetic acid (TCA). The homogenate was centrifuged at 10,000 rpm at 4°C for 10 min. Subsequently, 0.5 ml of the supernatant was added to 0.5 ml of a 0.1 M K-phosphate buffer (pH 7.0) and 1 ml of 1 M KI. The absorbance of the supernatant was measured at 350 nm. The content of H_2_O_2_ was calculated from a standard calibration curve that was prepared in 0.1% TCA at different H_2_O_2_ concentrations. The H_2_O_2_ concentration was expressed as μmol/g fresh weight (FW).

### Catalase Activity

Catalase activity in coleoptile segments that were prepared in the same manner as for the growth experiments was determined as described by Cavalcanti et al. (2004) with minor modifications. Briefly, after appropriate time of incubation 0.2 g coleoptile segment samples were homogenized in 1.5 ml of a 0.1 M K-phosphate buffer (pH 7.0). The homogenate was centrifuged at 20,000 rpm at 25°C for 20 min. Subsequently, 20 μl of the supernatant was added to 2 ml of 10 mM H_2_O_2_ and the decrease in absorbance was measured at 240 nm and 30°C. Enzyme activity was calculated using the molar extinction coefficient 36 × 10^3^/mM/m and expressed as μmol H_2_O_2_ oxidized/g FW/min.

### Determination of the MDA Content

Lipid peroxidation was determined by estimating the MDA content, which was determined in terms of the concentration of thiobarbituric acid-reactive substances (TBARS) as described by Hodges et al. (1999) with minor modifications. Coleoptile segments were preincubated for 1 h in control medium and immediately transferred to control medium with DHNQ at the appropriate concentration. IAA, at a final concentration of 100 μM, was included in the incubation medium when required. After appropriate time of incubation coleoptile segment samples of 0.5 g were immediately placed in liquid nitrogen. Then, the plant tissues were homogenized in 12.5 ml 80% ethanol. A 1 ml aliquot of the appropriately diluted sample was added to a test tube with 1 ml of either (1) a -TBA solution comprised of 20% (w/v) TCA and 0.01% butylated hydroxytoluene or (2) a +TBA solution containing the above plus 0.65% TBA. The samples were then mixed vigorously, heated at 95°C in a boiling water bath for 20 min, cooled and centrifuged at 10,000 rpm at 4°C for 10 min. The absorbance values were read at 440, 532, and 600 nm. MDA equivalents were calculated in the following manner:

1. [(Abs_532+TBA_) - (Abs_600+TBA_) - (Abs_532-TBA_ - Abs_600-TBA_)] = A
2. [(Abs_440+TBA_ - Abs_600+TBA_) 0.0571] = B
3. MDA equivalents (nmol/ml) = (A - B/157000) × 10^6^.

### Redox Activity

To determine the redox activity, which was estimated as the hexacyanoferrate III (HCF III) reduction, the coleoptile segments were prepared in the same manner as for the growth experiments. These coleoptile segments were then preincubated for 1 h in distilled water and immediately transferred to 1 mM Tris-HCl (pH 6.0) containing 0.5 mM CaCl_2_, 50 mM KCl and DHNQ at the appropriate concentration. IAA, at a final concentration of 100 μM, was included in the incubation medium when required. HCF III (ferricyanide), at a final concentration of 1 mM, was added to the incubation medium. The coleoptile segments were shaken at 100 rpm and the level of HCF III reduction was measured upon the addition of the coleoptile segments and every 30 min for the next 2 h. The redox activity, which was measured as the decay of the HCF III absorption, was monitored spectrophotometrically at 420 nm as was previously described by Federico and Giartosio (1983) and expressed in nmol of the reduced hexacyanoferrate III that was calculated per g of FW. Because naphthoquinones absorb the light at 420 nm, we modified the values of the HCF III reduction by the same as the ones that were recorded with no HCF III in the incubation medium. The results are the means of three independent experiments.

### Immunofluorescence Visualization of Cortical Microtubules

Cortical microtubules were fixed and immunostained as described earlier (Hejnowicz, 2005) with minor modifications. Briefly, the coleoptile segments were excised 3 mm below the tip of etiolated maize seedlings and incubated with naphthazarin, naphthazarin plus auxin and auxin, which were added to the incubation medium at the same time protocol as was described for the growth experiments. Subsequently, the samples were put on a glass slide covered by glue tape and were cut superficially with a razor blade. To preserve the current state of the tissue, they were submerged with a fixative (3.7% p-formaldehyde in a microtubule-stabilizing buffer, (MTSB), pH 6.8 with 1% dimethyl sulfoxide) for 1 h in a desiccator at room temperature. Afterward, the tissue samples were rinsed with MTSB twice and cut once more. The samples were incubated for 1.5 h with monoclonal anti-alpha-tubulin DM1A (Sigma-Aldrich Co.), diluted to 1:200 in a phosphate-buffered saline (PBS) (Nick et al., 1992) with bovine serum albumin (BSA) (1 mg/ml) in a humidity chamber at 37°C. When the incubation time was over, they were then rinsed in PBS and incubated again for 1.5 h with secondary antibodies against mouse IgG (FITC) at a dilution of 1:80 in order to visualize the α-tubulin. After incubation with the secondary antibody, the tissue samples were rinsed with PBS. The cortical microtubules were viewed using an Olympus IX81 Inverted Compound Microscope equipped with a FluoView FV1000 confocal system with an argon laser as the light source at an excitation wavelength of 488 nm (emission wavelength 500–600 nm). The collected photographs were further analyzed as z-stacks, which enabled over 50 cells per variant to be classified. To evaluate the orientation and anisotropy of the main microtubules, the concept of a nematic tensor from the physics of liquid crystal was used. Briefly, the direction of the gradient of the intensity of the secondary antibody signal enables the local direction that is normal for cortical microtubules to be defined (Uyttewaal et al., 2012; Burian et al., 2013). The quantitative data were obtained using ImageJ with an additional Fibril Tool plugin installed and the method described by Boudaoud et al. (2014) in which the circular average of the tangent direction defines the average orientation of the microtubules in this region and the circular variance of the tangent direction defines the score by assessing whether the microtubules are well ordered. According to Boudaoud et al. (2014), the following convention was used to analyze the anisotropy: 0 for no order of the microtubule arrays (purely isotropy) and 1 for perfectly ordered microtubule arrays (purely anisotropy).

### Statistical Analysis

The data were analyzed using Statistica software for Windows (STATISTICA data analysis software system, version 13.1^1^, United States) and MATLAB (The Mathworks, Natick, MA, United States) at a significance level of 0.05. One-way ANOVA was used to examine the statistical differences in the effect of naphthazarin on the IAA-induced coleoptile segments growth, pH medium changes and MT anisotropy. Afterward, the *post hoc* least significant difference (LSD) test was used for further analysis (*P* < 0.05). The Student’s *t*-test was used to evaluate the significance of the differences between the membrane potential values that were recorded at 0 and 60 min of the measurements. The correlation of the elongation growth and proton concentrations was calculated based on Spearman’s rank correlation. The analysis data for the microtubules – MT angle, mean MT angle and confidence limits – were analyzed based on the circular statistics. The Watson-Williams Test for Two-Sample was used to investigate the statistical differences between the microtubule arrays for all of the tested variants (Zar, 2010).

## Results

### The Effect of Naphthazarin (5,8-Dihydroxy-1,4-Naphthoquinone, DHNQ) on the Endogenous and IAA-Induced Growth of Coleoptile Segments

**FIGURE 1:**
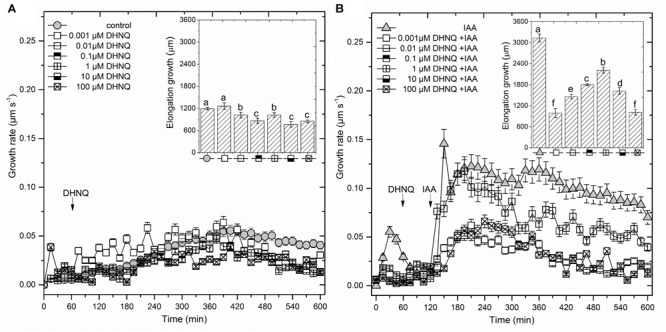
The effect of 5,8-dihydroxy-1,4-naphthoquinone (DHNQ) on the growth rate (μm s^-1^) of 1 cm-long maize coleoptile segments that had been incubated without **(A)** or with **(B)** IAA. The coleoptile segments were first preincubated (over 1 h) in a control medium, after which DHNQ in a range of concentrations from 10^-9^ to 10^-4^ M was added. IAA was added to the incubation medium at 2 h. The insets on the right side show the total elongation growth, which was calculated as the sum of the extensions from measurements at 3-min intervals over 10 h. Statistical analysis: Bars indicate means ± SEs. The data presented are the means of at least seven independent experiments. Mean values followed by the same letter are not significantly different from each other (LSD test, *P* < 0.05).

The addition of DHNQ to the control medium (1 h after the start of the experiment) inhibited the endogenous growth by *ca*. 15–40% (growth in the medium with no growth substances) of the maize coleoptile segments at almost all of the concentrations that were studied, excluding DHNQ at 0.001 μM (Figure 1A inset). For example, at the highest concentration (100 μM), naphthazarin reduced endogenous growth (1193 ± 32 μm, mean ± SE, n = 7) by *ca*. 30%, whereas at the lowest concentration (0.001 μM), it practically did not change it.

When auxin (IAA) was added to the control medium alone (2 h after the start of the experiment), it induced rapid growth with a maximal growth rate of *ca*. 0.12 μm/s. The kinetics of the IAA-induced growth rate of the coleoptile segments could be divided into two phases (biphasic kinetics); the first rapid phase, which took about 30 min, was followed by a long-lasting one. Interestingly, the first peak observed in the biphasic kinetics of the IAA-induced growth rate was abolished in the presence of all DHNQ concentrations, other than 1 μM DHNQ. In the presence of IAA, the total elongation growth of the maize coleoptile segments (calculated as the sum of the extensions that were measured at 3-min intervals over 10 h) amounted to 3122 ± 111 μm (mean ± SE, *n* = 7) (Figure 1B inset), and was approximately 2.5-fold greater than in the control medium. The data in Figure 1B (inset) indicates that DHNQ, when added after 1 h of the preincubation of the segments in the control medium, reduced the IAA-induced growth of the coleoptile segments at all of the concentrations that are studied. The dose–response curves that were constructed for the effect of DHNQ on the endogenous and IAA-induced elongation growth of the maize coleoptile segments (calculated as the sum of the extensions that were measured at 3-min intervals over 8 h between 120 and 600 min) differed in their shapes (Figure 3A). In the presence of IAA, the dose–response curve for the effects of DHNQ on the elongation growth of the maize coleoptile segments was bell-shaped with the maximum at 1 μM of DHNQ. However, in the case of the endogenous growth, the dose–response curve was practically linear. Interestingly, in the presence of IAA, naphthazarin at 0.001 μM and 100 μM reduced the elongation growth of the maize coleoptile segments to the same level, which was *ca*. 15% lower than the growth in the control medium (endogenous growth).

### Effect of DHNQ on Medium pH Measured Simultaneously With Growth

**FIGURE 2:**
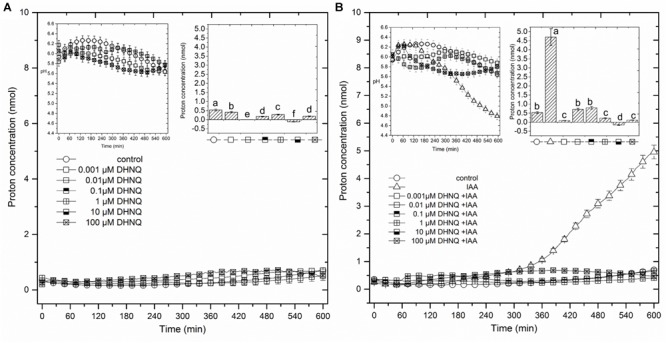
The effect of 5,8-dihydroxy-1,4-naphthoquinone (DHNQ) on the medium pH of the 1 cm-long maize coleoptile segments that had been incubated without **(A)** or with **(B)** IAA (the insets on the left side). The medium pH changes that were observed in the presence of all of the DHNQ concentrations that were studied are expressed as changes in the H^+^ concentration per coleoptile segment and are shown below the figure. The insets on the right side show the differences between the H^+^ concentration per coleoptile segment (nmol) at 600 and 120 min. Auxin and DHNQ were added to the incubation medium at the same time protocol as was described for the growth experiments. Statistical analysis: Bars indicate means ± SEs. The pH values (inset on the left) are the means of at least seven independent experiments, which performed simultaneously with growth. Mean values followed by the same letter are not significantly different from each other (LSD test, *P* < 0.05).

**FIGURE 3:**
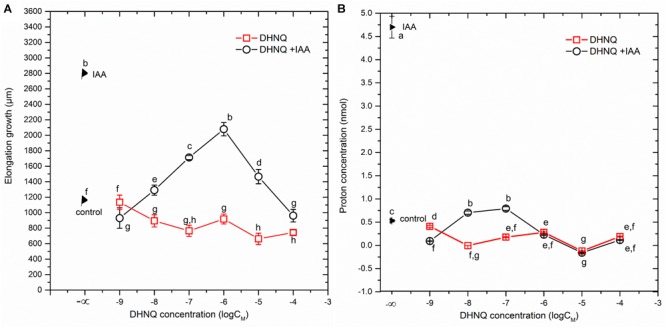
Dose–response curves for the effects of DHNQ on **(A)** an endogenous and IAA-induced growth of 1 cm-long maize coleoptile segments and **(B)** proton extrusion measured in the presence and absence of IAA. **(A)** The curves were constructed taking into account the total IAA-induced growth and endogenous growth, which was calculated as the sum of the extensions from measurements at 3-min intervals over 8 h between 600 and 120 min. **(B)** The curves were constructed taking into account the differences between the H^+^ concentration per coleoptile segment at 600 and 120 min. Statistical analysis: Bars indicate means ± SEs. Mean values followed by the same letter are not significantly different from each other (LSD test, *P* < 0.05).

The data that was obtained for medium pH, which was measured simultaneously with growth (Figure 2A), indicated that the coleoptile segments that had been incubated in the auxin-free medium changed its pH in a specific manner. Generally, within the first 2–3 h, an increase of pH to 6.0–6.3 was observed, followed by a slow decrease to a pH of approximately 5.6 after 10 h. When DHNQ was added to the control medium (after 1 h of the preincubation of the segments in the control medium), it diminished acidification of the medium (proton extrusion) at moderate (0.001, 0.01, 0.1, and 1.0 μM) concentrations and caused the alkalinization of the medium at higher ones (10 and 100 μM). When IAA was added to the control medium alone 2 h after the start of the experiment, an additional decrease in pH to *ca*. 4.8, compared to the control medium was observed. However, when DHNQ was added (after 1 h of the preincubation of the segments in the control medium), the IAA-induced proton extrusion decreased to the level that was observed in the control medium or to one that was significantly lower (Figure 2B). In order to express the pH changes in the medium much more suggestively, they are shown as Δ[H^+^] per coleoptile segment, where Δ[H^+^] means the difference between the H^+^ concentration ([H^+^]) at 600 and 120 min (addition of IAA) (Figures 2A,B, insets on the right side). The dose response curve for the effects of DHNQ on the proton extrusion of the maize coleoptile segments was sinusoidal when there was no IAA in the medium (Figure 3B). However, in the presence of IAA, the dose–response curve was linear with values that were lower than in the control medium.

### Effect of DHNQ on the Membrane Potential (*E*_m_) of Parenchymal Coleoptile Cells

The mean *E*_m_ of the parenchymal coleoptile cells that was measured in the control medium was -103.3 ± 1.1 mV (mean ± SE, *n* = 27). The addition of DHNQ to the control medium (after the stabilization of the *E*_m_) at the highest concentration (100 μM) caused a delayed (within 60 min) depolarization of the *E*_m_ by 25.7 mV, while at lower concentrations it depolarized *E*_m_ only slightly (DHNQ at 0.1, 1.0 and 10 μM) or not at all (DHNQ at 0.001 and 0.01 μM) (Table 1). The addition of IAA to the control medium alone produced characteristic changes in the membrane potential of the parenchymal cells: the initial, transient depolarization by *ca*. 6.0 mV (not shown here) was followed by a delayed hyperpolarization during which the potential was 14.8 mV more negative than the original value (-112.3 ± 6.1 mV, Table 1). When the coleoptile segments were first incubated with DHNQ for 60 min, and then IAA was subsequently added, the auxin-induced membrane hyperpolarization was eliminated at all of the concentrations that were studied, excluding DHNQ at 1 μM. In the case of the medium with 1 μM DHNQ, the addition of IAA led to the hyperpolarization of *E*_m_ by 9.5 mV, which was 35% less than that was observed with IAA alone (Table 1). Interestingly, DHNQ at 100 μM not only suppressed the IAA-induced hyperpolarization of the *E*_m_ but also caused an additional membrane depolarization (Table 1).

**Table 1:** *E*_m_ changes in the parenchymal coleoptile cells after the addition of 5,8-dihydroxy-1,4-naphthoquinone (DHNQ), IAA and DHNQ with IAA.

Treatments	*E*_m_ (mV)				
	A 0 min	B 20 min	C 40 min	D 60 min	Δ*E*_m_ (D-A) (mV)
0.001 μM DHNQ	–105.7 ± 2.7	–97.2 ± 6.1	–97.2 ± 7.1	–106.5 ± 6.7	–0.8a
0.01 μM DHNQ	–100.9 ± 5.1	–101.8 ± 5.4	–99.5 ± 8.1	–102.5 ± 8.8	–1.6a
0.1 μM DHNQ	–101.5 ± 5.3	–101.0 ± 2.3	–98.5 ± 7.5	–96.4 ± 9.6	5.1a
1 μM DHNQ	–102.1 ± 1.7	–103.7 ± 2.4	–97.3 ± 1.7	–95.8 ± 1.3	6.3*
10 μM DHNQ	–102.7 ± 2.6	–92.7 ± 2.8	–96.1 ± 2.8	–98.7 ± 4.6	4.0a
100 μM DHNQ	–107.1 ± 2.4	–100.0 ± 5.7	–93.9 ± 4.4	–81.4 ± 8.7	25.7*
100 μM IAA	–112.3 ± 6.1	–118.2 ± 5.4	–123.6 ± 2.8	–127.1 ± 7.4	–14.8*
0.001 μM DHNQ + IAA	–111.2 ± 5.9	–108.2 ± 3.3	–114.1 ± 10.	–107.3 ± 5.5	3.9a
0.01 μM DHNQ + IAA	–114.5 ± 5.5	–81.1 ± 6.7	–106.4 ± 6.9	–107.2 ± 6.7	7.3a
0.1 μM DHNQ + IAA	–99.0 ± 3.3	–99.6 ± 5.1	–81.4 ± 5.1	–100.1 ± 4.4	–1.1a
1 μM DHNQ + IAA	–102.7 ± 1.9	–106.4 ± 3.8	–111.9 ± 3.2	–112.2 ± 1.06	–9.5*
10 μM DHNQ + IAA	–102.4 ± 1.7	–105.6 ± 11.7	–102.9 ± 9.2	–109.4 ± 3.2	–7.0a
100 μM DHNQ + IAA	–67.3 ± 2.4	–64.8 ± 0.9	–61.8 ± 0.3	–60.9 ± 0.7	6.4*

*At time 0 (A), the control medium was exchanged for a new medium with the same salt composition but also containing DHNQ, IAA or DHNQ with IAA. In the case in which DHNQ was present together with IAA, the coleoptile segments were first preincubated for 1 h in the presence of DHNQ, whereupon the medium was exchanged for DHNQ plus IAA and the *E*_*m*_ was measured. The data are the means of at least six independent experiments. Error indicates ± SE. ^∗^Statistically significant (*P* < 0.05). ^*a*^Not statistically significant.*

### Effect of DHNQ on H_2_O_2_ Production and Catalase Activity

**FIGURE 4:**
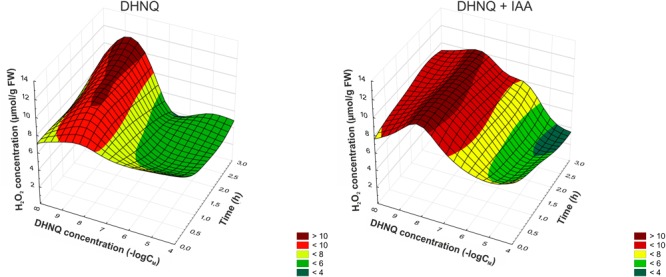
Dose–response curves for the effects of DHNQ and DHNQ plus IAA on the H_2_O_2_ level in the maize coleoptile segments as a function of time. The coleoptile segments were first preincubated for 1 h in the control medium, after which DHNQ at all of the concentrations tested was added. Time “0” indicates the H_2_O_2_ level after 1 h of incubation of the coleoptile cells in the presence of DHNQ. The hydrogen peroxide level in the control medium (without naphthoquinone) is shown here at a concentration of naphthazarin equal to log∞. IAA, at a final concentration of 100 μM, was applied at the same time protocol as DHNQ. The H_2_O_2_ level in the presence of IAA (without naphthoquinone) is shown here at a concentration of naphthazarin equal to log∞. The data are the means of at least seven independent experiments. The curves presented here are obtain by fitting data with use the distance-weighted least squares method.

Hydrogen peroxide production was increased with DHNQ concentrations lower than 0.1 μM during the 3 h of experiments, and the addition of IAA to the incubation medium 1 h after the addition of DHNQ accelerated this effect and extended it to higher concentrations (0.1 and 1 μM) of DHNQ. The application of DHNQ (after 1 h of the preincubation of the segments in the control medium) at concentrations higher than 0.1 μM resulted in a decrease in hydrogen peroxide production to values lower by almost 50% compared to the control after 3 h of experiments (7.7 ± 0.1 μmol g^-1^ FW, mean ± SE, *n* = 7) (Figure 4). Furthermore, when IAA was present in the control medium, this effect was intensified (especially at 10 and 100 μM DHNQ). The addition of IAA to the control medium alone (shown here as –log∞) enhanced H_2_O_2_ production only slightly (by 10%) compared to the control after 3 h of experiments.

**FIGURE 5:**
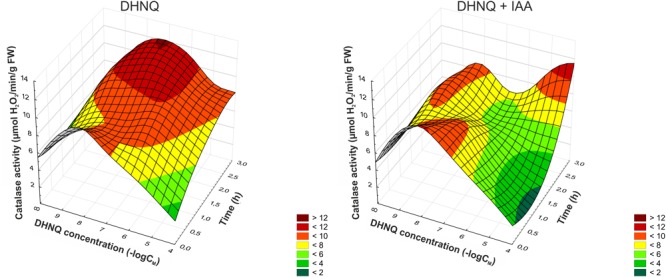
The effect of DHNQ and DHNQ plus IAA on the catalase (CAT) activity in the maize coleoptile cells as a function of time. The coleoptile cells were first preincubated for 1 h in the control medium, after which DHNQ at all of the concentrations that were studied was added. The catalase activity in the control medium (without naphthoquinone) or in the control medium with IAA are shown here at a concentration of naphthazarin equal to log∞. IAA, at a final concentration of 100 μM, was applied at the same time protocol as DHNQ. Statistical analysis: The data are the means of seven independent experiments. Bars indicate means ± SEs. The curves presented here are obtain by fitting data with use the distance-weighted least squares method.

Catalase plays a key role in the elimination of oxygen radicals by eliminating H_2_O_2_. The presence of the DHNQ in the incubation medium in almost all of the concentrations that were tested triggered an increase in the catalase activity (CAT) of the maize coleoptile segments compared to the control (5.13 ± 0.72 μmol H_2_O_2_ min^-1^g^-1^ FW, mean ± SE, *n* = 5, after 1 h) (Figure 5). Moreover, the addition of IAA to the incubation medium with DHNQ enhanced this effect (at 3–4 h) in the three concentrations that were tested: 0.001, 0.1, and 100 μM. Interestingly, the prolonged incubation time (to 3 h) of the coleoptile segments in the presence of low concentrations of DHNQ (lower than 1 μM) contributed to the growth of the CAT activity. This effect might be associated with the increased production of hydrogen peroxide (for a comparison, see Figures 4, 5). At the lowest concentration (0.001 μM) of DHNQ that was tested in the presence of IAA, the catalase activity (CAT) reached a value that was *ca*. two-fold greater than in the control medium (after 4 h).

### The Effects of DHNQ on Lipid Peroxidation

**FIGURE 6:**
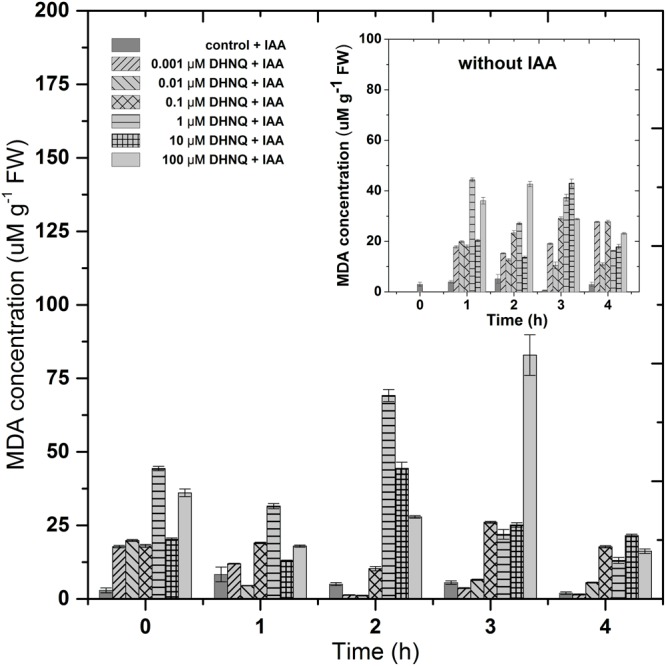
The impact of DHNQ and DHNQ plus IAA on the MDA content in the maize coleoptile cells as a function of time. The coleoptile cells were first preincubated for 1 h in the control medium, after which DHNQ at all of the concentrations that were tested was added. IAA, at a final concentration of 100 μM, was applied at time “0.” Statistical analysis: The data presented are the means of three independent experiments. Bars indicate means ± SEs.

Lipid peroxidation in the maize coleoptile cells that had been incubated with DHNQ and DHNQ and IAA, which was measured as the MDA concentration, is presented in Figure 6. The data that was obtained indicated that the addition of DHNQ to the control medium of the maize coleoptiles cells (after 1 h of preincubation) increased the MDA level at least four-fold (compared to the control; 2.92 ± 0.8 μM g^-1^ FW MDA) during first 3 h in all of the concentrations that were tested. The presence of DHNQ in the incubation medium elevated the MDA level in a concentration-dependent manner. At higher concentrations (over 1 μM), the lipid peroxidation status in the maize coleoptile cells exceeded 25 μM g^-1^ FW MDA after 3 h of incubation. The administration of IAA to the medium (after 1 h of preincubation with DHNQ) enhanced the MDA content to values that were higher than 25 μM g^-1^ FW MDA at the three highest concentrations: 1, 10 and 100 μM during the three first hours. The presence of IAA diminished the lipid peroxidation in the case of the lower concentrations of DHNQ (0.001 and 0.01 μM) to values of 1.53 ± 0.2 μM g^-1^ FW and 5.56 ± 0.2 μM g^-1^ FW, respectively, after 4 h of experiment.

### The Effects of DHNQ on the Redox Activity of the Coleoptile Segments

**FIGURE 7:**
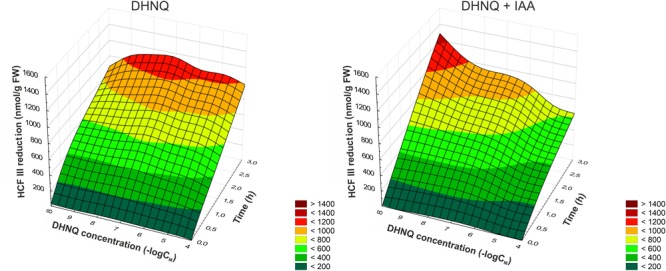
The effect of DHNQ and DHNQ plus IAA on the reduction of HCF III by the maize coleoptile cells as a function of time. The coleoptile cells were first preincubated for 1 h in distilled water and immediately transferred to an incubation medium containing DHNQ at the appropriate concentrations. IAA, at a final concentration of 100 μM, was, as required, included in the incubation medium. The level of the reduction of HCF III was measured after the addition of the coleoptile segments to the incubation medium every 30 min for the next 3 h. Statistical analysis: The data presented are the means of three independent experiments. The curves presented here are obtain by fitting data with use the distance-weighted least squares method.

The effect of DHNQ and DHNQ combined with IAA on the redox activity of maize coleoptile cells as a function of time is presented in Figure 7. The addition of DHNQ to the incubation medium increased the redox activity measured as HCF III reduction in all concentration tested only slightly as compared to the control. As can be seen in Figure 7, treatment of the maize coleoptile segments with DHNQ at a concentration of 0.1 μM stimulated the reduction of HCF III by ca. 30% after 3 h of the experiment, compared to the control (822.3 ± 19.6 nmol g^-1^ FW).

However, the addition of IAA to the incubation medium alone increase the redox activity of the maize coleoptile cells and reached a value of 1335.7 ± 39.7 nmol g^-1^ FW compared to the control. In the presence of IAA, at concentrations higher than 1 μM, DHNQ lowered the redox activity in the maize coleoptile segments within 3 h of the experiment. For example, treatment of the maize cells with 100 μM DHNQ and IAA induced an approximately two-fold decrease in the reduction of HCF III after 3 h compared to the control with IAA. The remaining DHNQ concentrations that were tested did not cause significant changes in the reduction of HCF III in the presence of IAA and reached values that were similar to the control conditions.

### The Effects of DHNQ on the Organization of the Cortical Microtubules

**FIGURE 8:**
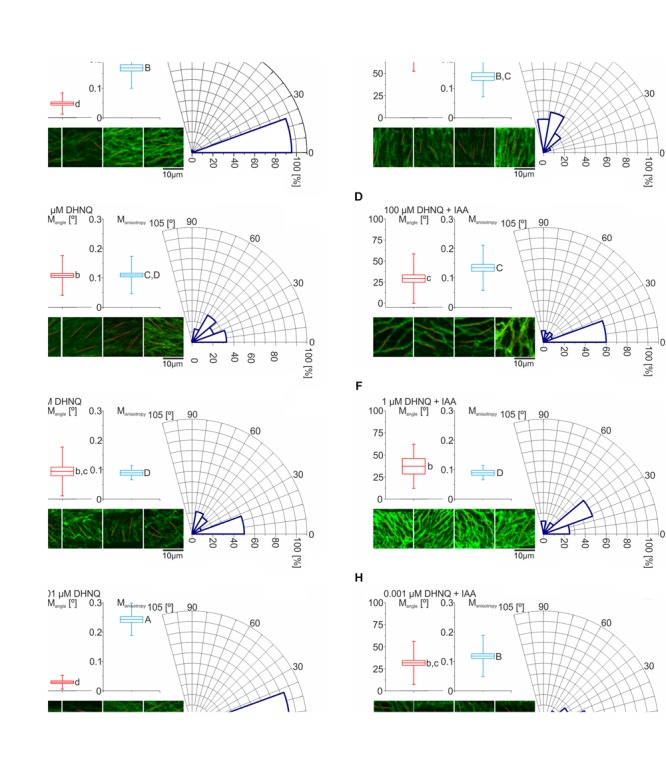
The effects of DHNQ on the organization of the cortical microtubules. **(A)** Control, **(B)** IAA, **(C)** 100 μM DHNQ, **(D)** 100 μM DHNQ with IAA, **(E)** 1 μM DHNQ, **(F)** 1 μM DHNQ with IAA, **(G)** 0.001 μM DHNQ, and **(H)** 0.001 μM DHNQ with IAA. To specify the mean angle and anisotropy of the cortical microtubules that had been stained with immunocytochemistry in the coleoptile epidermal cells of *Zea mays*, the FibrilTool was used. The excised coleoptile segments were incubated for 1 h in the presence of naphthazarin or naphthazarin and auxin. The control conditions for these variants were coleoptile segments that had been incubated in the control medium or the control medium with auxin. After incubation, the samples were cut lengthwise and fixed. The procedure for the fixation and staining of the samples was described in the section “Materials and Methods.” The photographs of four different cells with horizontal orientation that were collected with scale bars 10 μm, were analyzed in ImageJ with an additional FibrilTool plug installed, which gave direct information about the anisotropy of the cell system and the average angle of the cortical microtubule system. The average microtubule angle was transformed to be between 0° and 90°. The boxplots that were constructed for the cortical microtubule angle (M_angle_) and anisotropy (M_anisotropy_) show the mean and SE with the whiskers indicating the SD values. All M_angles_, their standard deviations and statistical analyses (based on The Watson-Williams Test for two samples) were performed on the basis of the circular statistic and the statistically significant differences between the variants are marked with a lowercase letter. In turn, the statistical analysis of anisotropy was based on an analysis of variance in Statistica (the *post hoc* test – NIR). The analysis of the variance [*F*-test, *F*(7.392) = 18.54] that show the differences between the variants are indicated with a capital letter. Boxes with the same letter do not differ significantly.

The cortical microtubule orientation of the maize coleoptile cells and its anisotropy in the presence of DHNQ and DHNQ with IAA are presented in Figure 8. The quantitative data were obtained using the FibrilTool and the method of Boudaoud et al. (2014), which was described above (see Material and Methods, Immunofluorescence visualization of the cortical microtubules). According to these authors, the following convention was used to analyze the anisotropy of the cortical microtubules: 0 for no order of the microtubule arrays (purely isotropy) and 1 for perfectly ordered microtubule arrays (purely anisotropy). The microtubule data were transformed to be between 0° (parallel to the long axis of the cell) and 90° (transverse to the long axis of the cell).

In the control conditions, the microtubule orientation was parallel to the long axis of the coleoptile cells with a mean angle (M_angle_) that was equal 7.1°± 5.4° (see Figure 8A). The responses of the maize coleoptile cells to the addition of IAA to the incubation medium are accompanied by explicit reorientations of the cortical microtubules. The average angle of the microtubules in relation to the long axis of the cell changes and assumes values that are 10-fold higher (M_angle_ = 70.2° ± 13.8°) (Figure 8B). However, in the presence of IAA, the difference of mean anisotropy (M_anisotropy_) of the two arrays was statistically insignificant (see Figures 8A,B).

In the presence of DHNQ at the concentrations 100 and 1 μM (Figures 8C,D) the cortical microtubules orientation was changed as compare to the control and the angle of the microtubules in relation to the long axis of the cell was ca. five-fold higher and achieved values: M_angle_ = 35.9° ± 2.2° and M_angle_ = 30.9° ± 12.5°, respectively. The anisotropy of cortical microtubules at these DHNQ concentrations was 0.11 ± 0.06 and 0.09 ± 0.05, respectively. The addition of auxin to the incubation medium containing DHNQ at 100 and 1 μM did not contribute to any significant changes, and both parameters: M_angel_ and M_anisotropy_ have reached similar values.

Interestingly, the lowest concentration of DHNQ (0.001 μM) had significantly different effect on the organization of cortical microtubules and its anisotropy. In this case, the orientation of cortical microtubules was parallel to the long axis of the cell and, moreover, it was different of the organization of microtubule in two higher concentrations of DHNQ (mentioned above). Furthermore, the anisotropy of microtubules was higher than in control condition reaching the highest value in the experiment (M_anisotropy_ = 0.24 ± 0.05). When auxin was present together with DHNQ at 0.001 μM the microtubule organization had changed to the similar as in the two higher concentrations tested by us and the angle of microtubules in relation to the long axis of the cell was approximately 30°. The arrangement of microtubules in the presence of auxin and DHNQ in the concentration of 0.001 μM also changed, decreasing to a lower value than in the control conditions (M_anisotropy_ = 0.12 ± 0.07).

Histograms of angles of cortical microtubule in relation to the long axis of the cell were shown also on Figure 8. The results obtained in the experiment were classified into three types of microtubule organization: longitudinal, where microtubule were oriented at 0–15°, oblique in which microtubules were oriented at 16–74° and transverse, where microtubules were oriented at 75–90°. As can be seen in Figure 8, the relative proportions of different microtubule arrays were similar in control and DHNQ 0.001 μM (Figures 8A,G), where the fraction of cells with longitudinal microtubule orientation was 95.4% and 93.5%, respectively. Treatment with DHNQ at higher concentrations (1 and 100 μM) and DHNQ at 0.001, 1, and 100 μM added together with IAA changed the histograms configurations and the oblique microtubule-arrays comprised the majority (Figures 8C–F,H). The fractions of transverse arrays were present in IAA where more than 55% of microtubules were oriented at 75–90° (Figure 8B).

## Discussion

In recent years, a great deal of attention has been paid to naturally occurring naphthoquinones as compounds that have a high level of biological activity and with remarkable structural properties. This high level of biological activity of naphthoquinones is based on two primary mechanisms: the first is the covalent modification of biological molecules at their nucleophilic sites in which quinones act as electrophiles, while the second consists in redox cycling in which ROS are generated (reviewed in El-Najjar et al., 2011; Kumagai et al., 2012; Klotz et al., 2014; Widhalm and Rhodes, 2016). The main goal of the experiments described here was to shed light on the toxic effect of naphthazarin (5,8-dihydroxy-l,4-naphthoquinone, DHNQ) on IAA-induced growth of plant cells. Understanding this effect is important for several reasons: the allelochemical properties of naphthoquinones and their effect on plant growth and development, the toxic effect of quinones on the environment as well as the possibility of using naphthoquinones as natural herbicides or pesticides (for example, see Akhtar et al., 2012; Chinchilla et al., 2017).

We found that the dose–response curves that were constructed for the effect of DHNQ on the endogenous and IAA-induced elongation growth of the maize coleoptile segments differed in shape (Figure 3A). In the presence of IAA, the dose–response curve was bell-shaped, while in the absence of IAA (endogenous growth), it was linear. Comparing the dose–response curves for the effect of DHNQ on IAA-induced elongation growth of maize coleoptile segments that were obtained here with ones that were obtained recently by us for 1,4-naphthoquinone (NQ) and 2-hydroxy-1,4-naphthoquinone (lawsone, NQ-2-OH) (Rudnicka et al., 2018), it should be pointed out that in the presence of IAA, they differ in shape. In the case of the dose–response curve that was constructed for the effects of 1,4-naphthoquinone and 2-hydroxy-1,4-naphthoquinone (lawsone) on IAA-induced growth, there were two extremes (a maximum at 10 μM and minimum at 0.1 and 1000 μM, respectively, Rudnicka et al., 2018), while for DHNQ, the dose–response curve was bell-shaped with a maximum at 1 μM. The different shapes of the dose–response curves that were observed for the effects of 1,4-naphthoquinone, 2-hydroxy-1,4-naphthoquinone and DHNQ on the IAA-induced elongation growth of the maize coleoptile segments probably resulted from their structural characteristics (such as the number and position of the hydroxyl groups) of the naphthoquinones. In order to shed light on the toxic effect of DHNQ on IAA-induced elongation growth of maize coleoptile segments, we first considered the interrelation between the elongation growth, proton extrusion and membrane potential (measured in the presence of IAA) which constitute the basis for the so-called “acid growth hypothesis” of auxin action in which PM H^+^-ATPase plays a key role (reviewed in Rayle and Cleland, 1992; Hager, 2003). In accordance with this hypothesis, at least in maize coleoptile segments, auxin causes the acidification of the cell wall (Rayle and Cleland, 1992) and the hyperpolarization of the membrane potential by stimulating the activity and/or the amount of the plasma membrane H^+^-ATPase. The acidification of the cell wall either directly lowers the yield threshold of the wall or optimizes the activity of the cell wall-localized proteins that loosen the wall, whereas the hyperpolarization of the membrane potential causes the activation of voltage-dependent, inwardly rectifying K^+^ channels, the activity of which contributes to the water uptake that is necessary for cell expansion (reviewed in Rayle and Cleland, 1992; Kutschera, 1994; Hager, 2003; Kutschera, 2006; Kutschera and Wang, 2016). The data in Figure 3 indicate that DHNQ, when added after 1 h of the incubation of the segments in the control medium, reduced the IAA-induced growth and proton extrusion of the coleoptile segments at all of the concentrations that were studied, thus suggesting that growth and the proton concentration are strongly correlated in the presence of DHNQ (Spearman’s rank correlation coefficient *r*_S_ = 0.73, *p* < 0.05). This finding is in good agreement with the “acid growth hypothesis” of auxin action and indicates that changes in IAA-induced growth of the maize coleoptile segments that were observed in the presence DHNQ might be mediated *via* PM H^+^-ATPase activity. This suggestion is also supported by the fact that naphthazarin eliminated the IAA-induced hyperpolarization of *E*_m_ (Table 1). There is no doubt that the IAA-induced plasma membrane hyperpolarization is a consequence of a stimulated proton extrusion through the H^+^-ATPase (Lohse and Hedrich, 1992; Rück et al., 1993; Hedrich et al., 1995). Moreover, the first peak in the biphasic kinetics of IAA-induced growth (Figure 1B), which is attributed to the PM H^+^-ATPase activity (Becker and Hedrich, 2002) was abolished in the presence of all DHNQ concentrations, excluding DHNQ at 1 μM.

The higher elongation growth in the presence of 1 μM of DHNQ may probably be related with the fact that ROS might be involved in the chemorheological wall-loosening reaction that is responsible for the auxin-controlled growth of maize coleoptiles (Frahry and Schopfer, 2001; Schopfer, 2001; Schopfer et al., 2002). In turn, the much greater inhibitory effect of DHNQ on the IAA-induced growth of maize coleoptile cells compared to that of NQ-2-OH and NQ (Rudnicka et al., 2018) probably results from the differences in the structure of the molecule and the higher content of hydroxyl groups in naphthazarin. For the lower toxicity of NQ-2-OH compared to DHNQ, the substitution of the hydroxyl group at the C_2_ position (lawsone) is also important. It contributes to reducing the electrophilicity C_3_, and moreover, causes a steric hindrance in the interactions with the nucleophiles (Ollinger and Brunmark, 1991; Klotz et al., 2014). A steric hindrance effect was previously proposed by Kot et al. (2010) in order to explain the higher toxicity of 5-hydroxy-1,4-naphthoquinone (juglone) on the urease activity of the jack bean compared to lawsone.

Plant cell growth is connected with many intracellular interactions but also with the organization of the cortical microtubules and cellulose microfibrils, which can dynamically respond to environmental factors such as growth regulators, light or mechanical stimulus (Zandomeni and Schopfer, 1993, 1994; Cyr and Palevitz, 1995; Chan, 2011). In order to understand the influence of DHNQ on the auxin-induced growth of plant cells, our next step was to investigate their impact on microtubule orientation. It was found that the application of IAA to the incubation medium that contained excised coleoptile segments reversed the longitudinal orientation of the cortical microtubules into a transverse orientation (Zandomeni and Schopfer, 1993; Fischer and Schopfer, 1997; Schopfer and Palme, 2016). Our results clearly demonstrate that at all of the concentrations that were tested naphthazarin changed the auxin-induced reorientation of the cortical microtubules (mentioned above) from perpendicular to oblique with respect to the long cell axis (Figure 8). This effect may be related to the nucleophilic interaction of naphthoquinone with the proteins that are associated with the cortical microtubule reorientation processes. Kirik et al. (2012) found that TON2 [a homolog of maize DCD1 and ADD1 (Wright et al., 2009)], which is a subunit of protein phosphatase 2A, acts as a regulator of the microtubule nucleation geometry, thus encouraging the formation of branch nucleation in experiments with *A. thaliana*. This formation allows a greater proportion of the discordant angles of microtubules and promotes the transition of the array order. A dysfunction of the TON2 subunit leads to a strong reduction in the branching frequency, and subsequently, affects the correct microtubule reorientation in response to a stimulus. A similar effect was found in maize coleoptile cells that had been incubated in the presence of all of the concentrations of DHNQ and IAA (see Figure 8), in which the microtubule orientation was oblique despite the addition of IAA. These data significantly differ from the research that was performed on animal cells by Acharya et al. (2011) in which naphthazarin caused microtubule depolymerization, which was not observed in our experiments. Acharya et al. (2011) found that human non-small lung epithelial carcinoma (A549) cells that were treated with varying concentrations of naphthazarin (1–25 μM) showed that this naphthoquinone depolymerizes the interphase microtubules and inhibits tubulin polymerization in a dose-dependent manner. Moreover, DHNQ changed the organization of the spindle microtubules from bipolar to multipolar at 10 μM or totally eliminated the formation of spindles at 25 μM.

As a stress factor, naphthazarin can increase the production of ROS, which can interfere with different cellular processes, thus causing an inhibition of growth in plant cells (Bais et al., 2003; Fujita et al., 2006; Chinchilla et al., 2017). The data presented in Figure 4 demonstrate that at lower concentrations (<0.1 μM) DHNQ enhanced the H_2_O_2_ production in maize coleoptile cells, which was accelerated and extended in the presence of IAA. These results closely correlate with the catalase activity (CAT), which is presented in Figure 5. CAT is a key enzyme that decomposes H_2_O_2_ into water and molecular oxygen and that is involved in the removal of toxic peroxides. At lower concentrations of DHNQ, CAT activity is increased and may cause an accumulation of H_2_O_2_ (Figure 4). The treatments of maize coleoptile segments with DHNQ at almost all of the concentrations that were studied induced lipid peroxidation (Figure 6) and increased the MDA content compared to the controls. The presence of IAA decreased the MDA concentrations, which might suggest that exogenous auxin plays a role in lipid peroxidation (Malenčić et al., 2012; Piotrowska-Niczyporuk and Bajguz, 2014).

Quinones have the ability to affect the plasma membrane redox systems, which in plant cells is connected with the depolarization of the plasma membrane, proton release and elongation growth (Lüthje et al., 1997). The data that was obtained in our experiments show that almost all of the concentrations of DHNQ that were studied were active in the stimulation of the reduction of HCF III by maize coleoptile cells (except for DHNQ at 100 μM) (Figure 7). The results presented here also show that the presence of IAA slightly decreased the reduction of HCF III that is induced by DHNQ in maize coleoptile segments compared to the variant without auxin. When IAA was added to the incubation medium alone, it stimulated a reduction in HCF III as was previously shown by Lekacz and Karcz (2006). It may be proposed that auxin contributes to the weakening of the interaction between the plasma membrane components, and subsequently, changes the fluidity of the lipid system (de Melo et al., 2004; Celik et al., 2006). This phenomenon can decrease the HCF III reduction in the presence of DHNQ later by disturbing the integration of naphthoquinone into the plasma membrane according to the hypothesis that was proposed by Lüthje et al. (1992). Comparing the redox activity of the DHNQ that was obtained here with a reduction of HCF III by maize coleoptile segments with the ones that were obtained recently by us for 1,4-naphthoquinone and 2-hydroxy-1,4-naphthoquinone (lawsone) (Rudnicka et al., 2018), it should be emphasized that naphthazarin is less active in redox cycling. Considering the information mentioned above, it may be proposed that the plasma membrane redox system are only slightly involved in a toxic effect of DHNQ on the IAA-induced growth of maize coleoptile cells.

To summarize, naphthazarin, which is a well-known secondary metabolite, has a toxic effect on the auxin-induced growth of maize coleoptile cells. This impact may be associated with both the direct and indirect interactions of naphthoquinone with the components of plant cells. It might be suggested that naphthazarin may directly inhibit the PM H^+^-ATPase activity *via* an arylation process where it reacts with the thiol groups of the protein or indirectly by producing ROS *via* redox cycling. Although we did not measure directly PM H^+^-ATPase activity our hypothesis that DHNQ inhibit the PM H^+^-ATPase activity is supported by three facts: (1) the diminished or eliminated IAA-induced proton extrusion, (2) the suppressed IAA-induced hyperpolarization of the *E*_m_ and (3) the abolished first peak in the biphasic kinetics of the IAA-induced growth rate (excluding DHNQ at 1 μM). In addition, our data, showing that DHNQ at 0.001 and 100 μM reduced the IAA-induced elongation of the coleoptile segments to the same level (bell-shaped dose–response curve) might suggest that at low DHNQ concentrations (<0.1 μM) ROS may play a role in a toxic effect of DHNQ on IAA-induced growth. Taking into account the findings obtained by other authors, it can be also suggested that naphthazarin, because of its structural similarity to juglone (5-hydroxy-1,4-naphthoquinone) can affect the auxin nuclear signaling system or interfere with the cytoplasmic auxin signaling mechanisms, which consist of ABP1 (auxin binding protein 1), TMK (transmembrane kinase) and ROPs (Rho-like GTPase) (Woo et al., 2002; Tian et al., 2003; Shishova and Lindberg, 2010; Xu et al., 2010, 2014; Nagawa et al., 2012; Grones et al., 2015). Another possibility is that ROS-induced cytosol Ca^2+^ elevations may inhibit the PM H^+^-ATPase activity and depolarize the plasma membrane potential (Kinoshita et al., 1995; Polevoi et al., 1996; Brault et al., 2004; Mori and Schroeder, 2004; Trouverie et al., 2008). Changes in the cytosol Ca^2+^ level also has an impact on microtubule organization. A distribution in the microtubule organization can provoke changes in the phosphorylation status of the microtubule proteins (Cyr and Palevitz, 1995) or by loss of function of microtubules proteins subunits, e.g., TON2 (Camilleri et al., 2002; Kirik et al., 2012).

Taking above into account it is suggested that naphthazarin with its broad spectrum modes of action could be considered as a component of new, safer bioherbicides and biopesticides.

## Author Contributions

WK and MR planned and designed the research. MR and ML performed the experiments, the statistical analyses, and interpretation of experimental results. MR wrote the manuscript. WK revised the manuscript. All authors have read and approved the submission of the manuscript to frontiers in Plant Science.

## Conflict of Interest Statement

The authors declare that the research was conducted in the absence of any commercial or financial relationships that could be construed as a potential conflict of interest.

## Footnotes

1 www.statsoft.pl
